# Genetic mutations and molecular mechanisms of Fuchs endothelial corneal dystrophy

**DOI:** 10.1186/s40662-021-00246-2

**Published:** 2021-06-15

**Authors:** Xuerui Liu, Tao Zheng, Chuchu Zhao, Yi Zhang, Hanruo Liu, Liyuan Wang, Ping Liu

**Affiliations:** 1 Department of Ophthalmology, The First Affiliated Hospital of Harbin Medical University, Harbin, Heilongjiang China; 2 The Beijing Institute of Ophthalmology, Beijing Tongren Hospital, Capital Medical University, Beijing, China

**Keywords:** Fuchs endothelial corneal dystrophy, Genetic mutations, Mechanisms, Therapy

## Abstract

**Background:**

Fuchs endothelial corneal dystrophy is a hereditary disease and the most frequent cause of corneal transplantation in the worldwide. Its main clinical signs are an accelerated decrease in the number of endothelial cells, thickening of Descemet’s membrane and formation of guttae in the extracellular matrix. The cornea’s ability to maintain stromal dehydration is impaired, causing painful epithelial bullae and loss of vision at the point when the amount of corneal endothelial cells cannot be compensated. At present, apart from corneal transplantation, there is no other effective treatment that prevents blindness.

**Main text:**

In this review, we first summarized the mutations of *COL8A2*, *TCF4*, *TCF8*, *SLC4A11* and *AGBL1* genes in Fuchs endothelial corneal dystrophy. The molecular mechanisms associated with Fuchs endothelial corneal dystrophy, such as endoplasmic reticulum stress and unfolded protein response pathway, oxidative stress, mitochondrial dysregulation pathway, apoptosis pathway, mitophagy, epithelial-mesenchymal transition pathway, RNA toxicity and repeat-associated non-ATG translation, and other pathogenesis, were then explored. Finally, we discussed several potential treatments related to the pathogenesis of Fuchs endothelial corneal dystrophy, which may be the focus of future research.

**Conclusions:**

The pathogenesis of Fuchs endothelial corneal dystrophy is very complicated. Currently, corneal transplantation is an important method in the treatment of Fuchs endothelial corneal dystrophy. It is necessary to continuously explore the pathogenesis of Fuchs endothelial corneal dystrophy and establish the scientific foundations for the development of next-generation corneal therapeutics.

## Background

Fuchs endothelial corneal dystrophy (FECD) is a genetically heterogenous disease accompanied by irreparable damage to the corneal endothelium [1]. FECD can occur via autosomal dominant inheritance, but it is usually a sporadic disease [2, 3]. In 2016, FECD accounted for 36% of corneal transplantation in United States [4]. FECD is characterized by a thickening of Descemet’s membrane (DM) and the appearance of guttae [5]. The accelerated loss of corneal endothelial cells (CECs) first appears in the center of the cornea, and the same clinical signs appear on the periphery of the cornea. The mosaic defect of the corneal endothelium due to cell loss causes living cells to respond by proliferating and migrating, resulting in abnormalities in uniform size (polymegathism) and variations in hexagonal shape (pleomorphism) [6, 7].

The most recent International Classification of Corneal Dystrophies categorizes FECD into two types: 1) rare early-onset FECD, and 2) more common late-onset FECD. Early-onset FECD, ascribed to mutations in the collagen type VIII alpha 2 chain (*COL8A2,* MIM 12052) [8], usually begins in the first decade of life. On average, late-onset FECD manifests in the fifth decade of life and implicates rare mutations in solute carrier family 4 sodium borate transporter member 11 (*SLC4A11*, MIM 610206) [9, 10], transcription factor 8 gene (*TCF8*, MIM 189909) [11], transcription factor 4 gene (*TCF4*, MIM 602272) [12], lipoxygenase homology domains 1 (*LOXHD1*, MIM 613072) [13] and ATP/GTP binding protein like 1 (*AGBL1*, MIM 615496) [4].

The emerging clinical technologies prompted us to learn more about the clinical manifestations of FECD. As the disease develops, the epithelium does not change significantly during the early stage to the painful epithelial bullae caused by damage to the corneal pump function [1]. Confocal microscopy reveals that the Bowman layer is bright and reflective, with increased reflectivity in the basal epithelial and anterior stromal layers in FECD patients [14]. The collagen fibers in the posterior stroma with fewer fibrous connections are looser than in the anterior stroma. This corneal edema leads to an increase in the posterior corneal hydration, causing the posterior stroma to swell to the anterior chamber and the central cornea to thicken [15]. In normal corneal tissue, DM comprises two layers and is secreted by CECs. In adults, an anterior banded layer has a constant thickness of 3 μm, and a posterior non-banded layer has an approximate thickness of 10 μm throughout its lifespan [16]. Guttae, the focal nodules in DM, are deposited in the center of the cornea, spreading out from this point in FECD. As the density of endothelial cells decreases, the shape and size of the endothelial cell changes. Overall, having a better understanding of FECD’s genetic mutations and molecular mechanisms provides us with unique insights into FECD development and potential treatment options. The purpose of this article is to review gene mutations, molecular mechanisms and future therapies of FECD, which form the basis and rationale for the proposed management for FECD.

## Main text

### FECD genetic mutations

#### *COL8A2* gene mutations in FECD

The *COL8A2* gene is found on chromosome 1; it encodes the α2 chain of short-chain collagen VIII, which is an extracellular matrix (ECM) protein and constitutes a major component of DM [8]. The *COL8A2* gene is closely related to early-onset FECD. Here, the substitution of glutamine for lysine is caused by a point mutation (p.Q455K) that was traced to an English family lineage [17]. Subsequently, a study of FECD patients showed a leucine-to-tryptophan substitution (p.L450W) in *COL8A2* [8]. A study that genotyped FECD from Korean patient revealed a glutamine-to-valine (p.Q455V) variation, which was projected to disrupt the interplay between *COL8A2* and *COL8A1* [18]. Mutations of the *COL8A2* found in recent FECD studies are shown in Table 1 [8, 17–21].

**Table 1:** Mutations of *COL8A2* gene on chromosome 1p34.3 and changes in its protein domain

Gene	Nucleotide change	Amino acid change	References
*COL8A2*	c.464G > A	p.R155Q	[17–20]
	NA	p.R304Q	[17, 19]
	NA	p.R434H	[17, 19]
	NA	p.Q455K	[17, 19]
	NA	p.G357R	[17, 19]
	NA	p.P575L	[17, 19]
	c.1370-1371CA > GT	p.Q455V	[18, 19]
	c.105G > A	p.A35A	[18–20]
	c.1485G > A	p.G495G	[18–20]
	c.1505C > T	p.T502M	[18, 20]
	NA	p.G3R	[19]
	c.1330 T > C	p.A441A	[19]
	c.1349 T > G	p.L450W	[8, 19]
	NA	p.P486P	[19]
	c.1610G > A	p.D537N	[19]
	c.1643A > G	p.N548S	[19]
	NA	p.P586P	[19]
	c.1951G > A	p.Y648Y	[19]
	c.1005C > G	p.L335L	[20]
	c.1526C > A	p.P508P	[21]
	c.1491G > A	p.A497T	[21]

*NA* not available

#### *TCF4* gene mutations in FECD

The *TCF4* gene, also known as *E2–2*, is found on chromosome 18 and encodes the E2–2 protein, which belongs to a family of basic helix-loop-helix transcription factors. *TCF4* plays a valuable role in many developmental processes and is related to transforming growth factor-β (TGF-β) signaling pathways and epithelial-mesenchymal transition (EMT) and programmed cell death [22–24]. *TCF4* gene mutation is the leading factor causing FECD. A genome-wide association study analysis revealed four single-nucleotide polymorphisms (SNPs) (rs17595731, rs613872, rs9954153, and rs2286812) in *TCF4* that were independently correlated with FECD [12]. Subsequent studies confirmed that *TCF4* (rs613872) has a significant correlation with FECD [25–28]. Of note, a recent genome-wide association study reported that *TCF4* SNP rs784257 was the most influential SNP in FECD among the discovery specimens; it had a strong correlation imbalance with rs613872 [29]. Another study, the results of which remain to be verified, reported that a SNP was situated in close proximity to the CTG repeat sequence [30]. Moreover, the genome-wide association study identified three novel loci meeting genome-wide significance (*P* < 5 × 10^−8^): *KANK4* rs79742895, *LAMC1* rs3768617 and *LINC00970/ATP1B1* rs1200114 [29]. Interestingly, the mutated genes were sex-specific, with *LAMC1* mutation at a higher risk in women and *TCF4* mutation at a higher risk in men [29].

*TCF4* was first found to be caused by the CTG trinucleotide repeat (TNR) amplification in the third intron [31]. Wieben et al.’s study showed that 79% of the Caucasian FECD patients carried repeat lengths of more than 50. In contrast, there were usually only less than 40 repeats in unaffected individuals included in the study. Using genotyping, CTG18.1 allele amplified in a Chinese population was found to have a strong correlation with FECD. Indeed, it may be the main pathogenic variant of FECD in this population [32]. In the Caucasian population, the severity of the disease may be directly related to the repeat length, whereas no such association was found in a Japanese cohort [33–35]. Although the correlation between CTG repeat amplified polymorphism and FECD is stronger than that of the *TCF4* rs613872 polymorphism, the combination of the two may better predict susceptibility to FECD [27].

#### *TCF8* gene mutations in FECD

The *TCF8* gene, also called the zinc finger E-box binding homeobox 1 (*ZEB1,* MIM 189909), is situated on chromosome 10 and encodes the ZEB1 protein, which can be up-regulated by *TCF4* expression [36]. It restrains collagen I expression and mediates EMT; here, the characteristics of epithelial cells disappear, such as cell-cell interaction, and migration and mesenchymal phenotypes are gained [24]. A study examining *TCF8* variants in FECD patients (55 women, 19 men) in China found that heterozygous mutations (p.N696S) in *TCF8* were present in only one of the patients [37]. Subsequently, another study on a FECD cohort of Caucasian adult males and females identified five missense mutations (p.N78T, p.Q810P, p.Q840P, p.A905G, and p.P649A) in *TCF8*; a change of one single nucleotide caused a different amino acid to be inserted into the resulting protein [11]. The mutations of the *TCF8* gene found in recent FECD studies are shown in Table 2 [11, 19, 37–39].

**Table 2:** Mutations of *TCF8* (*ZEB1*) gene on chromosome 10p11.22 and changes in its protein domain

Gene	Nucleotide change	Amino acid change	References
*TCF8* (*ZEB1*)	c.2522A > C	p.Q841P	[38]
	c.619A > G	p.S207G	[38]
	c.192T > C	p.D64D	[37, 39]
	NA	p.T232T	[39]
	c.2197G > A	p.E733K	[39]
	c.2453C > T	p.A818V	[39]
	c.2840 T > A	p.L947stop	[39]
	NA	p.S234S	[39]
	c.2519A > C	p.Q840P	[11, 39]
	c.2087A > G	p.N696S	[37]
	c.232A > G	p.N78T	[11]
	c.1945C > G	p.P649A	[11]
	c.2429A > C	p.Q810P	[11]
	c.2714C > G	p.A905G	[11]
	c.666 T > C	p.S201S	[19]
	c.852 T > C	p.S263S	[19]
	c.1721A > G	p.K553R	[19]
	c.1738C > T	p.P559S	[19]
	c.2037C > G	p.N658K	[19]
	c.2124A > C	p.P687P	[19]
	c.2623C > A	p.Q854K	[19]

*NA* not available

#### *LOXHD1* gene mutations in FECD

The *LOXHD1* gene is situated on chromosome 18 and encodes the LOXHD1 protein, which is believed to direct proteins to the plasma membrane [40]. *LOXHD1* was initially related to human autosomal recessive and progressive hearing loss [41, 42]. A missense mutation (p.R547C) in *LOXHD1* was first confirmed in three large families with FECD. Moreover, a study on a cohort of sporadically affected individuals revealed 14 additional nonsynonymous coding variants and a missense variant (p.L635P); these were absent from control chromosomes [13]. The mutations of the *LOXHD1* gene found in recent FECD studies about are shown in Table 3 [13, 38].

**Table 3:** Mutations of *LOXHD1* gene on chromosome 18q21.1 and changes in its protein domain

Gene	Nucleotide change	Amino acid change	References
*LOXHD1*	c.5272A > T	p.T1758S	[13]
	c.1904 T > C	p.L635P	[13]
	NA	p.D53E	[13]
	NA	p.S81N	[13]
	c.469C > T	p.R157C	[13]
	NA	p.R524C	[13]
	c.1639C > T	p.R547C	[13]
	c.1759C > T	p.R587W	[13]
	c.1945G > A	p.D649N	[13]
	c.2251C > T	p.R751W	[13]
	NA	p.R787C	[13]
	NA	p.L1292F	[13]
	NA	p.E1742K	[13]
	c.5395C > T	p.R1800W	[13]
	NA	p.E1985Q	[13]
	NA	p.H2038N	[13]
	c.6413G > A	p.R2138Q	[38]
	c.3463A > G	p.R1155G	[38]
	c.6107 T > C	p.A2036V	[38]

*NA* not available

#### *SLC4A11* gene mutations in FECD

The *SLC4A11* gene is situated on chromosome 20 and encodes the protein SLC4A11, which is usually situated on the cell surface and performs membrane transport functions (OH^−^/H^+^/NH_3_/H_2_O) [43–45]. *SLC4A11* is associated not only with FECD but with other types of corneal dystrophy, such as congenital hereditary corneal dystrophy type 2 and Harboyan syndrome [46]. In 2008, three missense variations (p.E399K, p.G709E and p.T754M) and a deletion variation (c.99-100delTC) that did not show up in matched controls were confirmed in FECD patients from India and China- all the variants are presumed to be pathogenic mutations [47]. The study also reported 15 non-pathogenic mutations (ten silent mutations and five missense mutations) [47]. In 2010, seven heterozygous missense novel variations (p.E167D, p.R282P, p.G583D, p.G742R, p.Y526C, p.V575M and p.G834S) resulting in the pathogenesis of adult FECD were identified in a study of 192 sporadic cases and a three-generation family [9]. The mutations of the *SLC4A11* gene found in recent FECD studies are shown in Table 4 [9, 19, 20, 47–49].

**Table 4:** Mutations of *SLC4A11* gene on chromosome 20p13 and changes in its protein domain

Gene	Nucleotide change	Amino acid change	References
*SLC4A11*	c.501G > C	p.E167D	[9]
	c.845G > C	p.R282P	[9]
	c.1577A > G	p.Y526C	[9]
	c.1723G > A	p.V575M	[9]
	c.1748G > A	p.G583D	[9]
	c.2224G > A	p.G742R	[9]
	c.2500G > A	p.G834S	[9]
	c.497A > G	p.N150S	[19]
	c.522C > T	p.R158R	[19]
	c.1437G > A	p.T463T	[19]
	c.2232G > A	p.H728H	[19]
	c.2706C > T	p.D886D	[19]
	c.1659C > T	p.N553N	[19]
	c.1195G > A	p.E399K	[47]
	c.2126G > A	p.G709E	[47, 48]
	c.2261C > T	p.T754M	[47]
	c.99-100delTC	p.S33SfsX18	[47]
	c.405G > A	p.A135A	[19, 47]
	c.481A > C	p.R161R	[19, 47]
	c.639G > A	p.S213S	[19, 20, 47]
	c.951G > A	p.T317T	[47]
	c.1179C > T	p.F393F	[47]
	c.1215C > T	p.I405I	[47]
	c.1620C > T	p.L540L	[47]
	c.1938G > A	p.A646A	[47]
	c.2499G > A	p.T833T	[19, 20, 47]
	c.215A > C	p.N72T	[47]
	c.271A > G	p.M91V	[47]
	c.1694C > T	p.S565L	[47]
	c.719G > C	p.W240S	[48]
	c.1304C > T	p.T434I	[48]
	c.1519G > A	p.V507I	[48]
	c.2027A > G	p.Q676R	[49]
	c.2195dupT	p.L732fs	[49]
	c.1237G > A	p.G413R	[49]
	c.2263C > T	p.R755W	[49]
	NA	p.R869H	[49]

*NA* not available

#### *AGBL1* gene mutations in FECD

The *AGBL1* gene, also called cytosolic carboxypeptidase 4, is situated on chromosome 15 and encodes the AGBL1 protein [49]. A genome-wide linkage scan of 92 individuals from 22 families with FECD revealed that chromosome 15 was related to FECD [50]. Sequencing of *AGBL1* confirmed a causal nonsense mutation (p.R1028*) in a three-generation FECD family. Further sequencing of two FECD-affected cases confirmed this result, and another missense mutation (p.C990S) was found in three unrelated individuals (see Table 5) [51]. Further, an immunoprecipitation assay suggested that AGBL1 protein bound to TCF4 but not to TCF8-a mutation of AGBL1 significantly reduced binding affinity to TCF4 [51].

**Table 5:** Mutations of *AGBL1* gene on chromosome 15q25.3 and changes in its protein domain

Gene	Nucleotide change	Amino acid change	References
*AGBL1*	c.2969G > C	p.C990S	[51]
	c.3082C > T	p.R1028*	[51]

*indicates that the mutation causes the stop codon to appear prematurely at position 1028

### Molecular mechanisms

#### Endoplasmic reticulum stress and unfolded protein response pathway

**Fig. 1:**
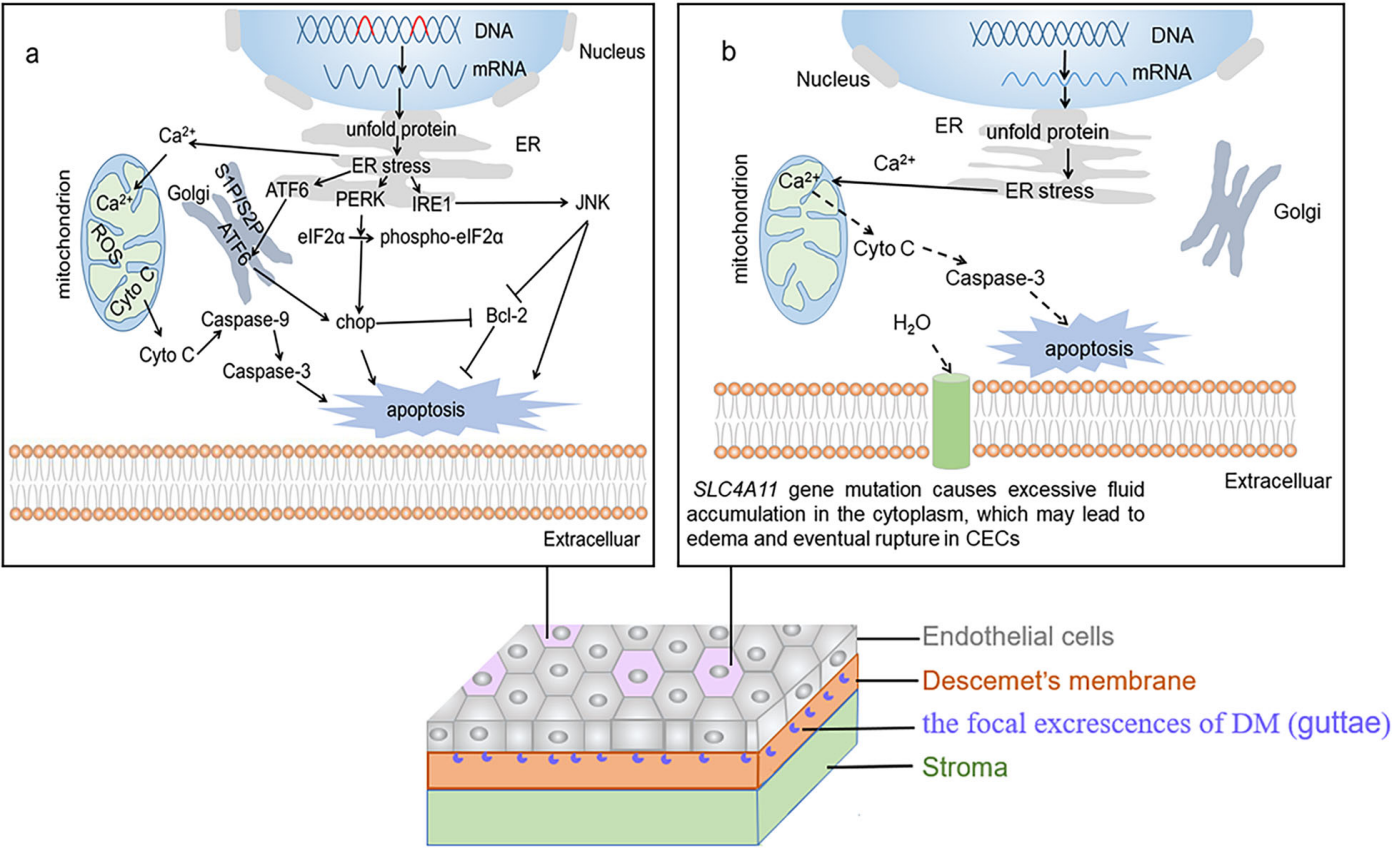
Possible pathways leading to the loss of FECD CECs. **a** Gene mutations lead to the accumulation of unfolded proteins, which continue to activate ER stress, and further induce apoptosis through the three UPR pathways (ATF6, PERK, IRE1). Meanwhile, sustained ER stress can induce cell apoptosis through the mitochondria. **b** Ca^2+^ overload in FECD CECs may lead to apoptosis and *SLC4A11* mutations are likely to result in CECs edema and rupture. eIF2α: α-subunit of eukaryotic translation initiation factor 2; JNK: c-Jun N-terminal kinase; S1P: site-1 protease; S2P: site-2 protease. The pieces of DNA in red represent the missense mutations of *COL8A2* and/or *SLC4A11* and/or *LOXHD1*. The purple cells represent dysfunctional CECs in FECD. The blue moons represent the guttae-the focal excrescences of DM

Proper protein folding is critical to overall cellular functioning. Cells have a conservation mechanism in the endoplasmic reticulum (ER) that allows them to avoid protein misfolding and deal with cytotoxic misfolded proteins, of which the excessive accumulation results in ER stress. Unfolded protein response (UPR) is a pro-survival response; it reduces unfolded protein accumulation and restores normal ER function. However, if protein aggregation persists and stress cannot be resolved, the signal changes from pro-survival to pro-apoptotic. COL8A2 accumulates in the ER of the corneal endothelia of FECD patients with L450W *COL8A2* mutations [52]. Two studies confirmed that a *COL8A2* transgenic knock-in mouse model of FECD exhibited dilated rough ER, overexpression of UPR-associated genes and proteins and UPR-associated apoptosis [53, 54]. A subsequent study confirmed that the missense mutations of *SLC4A11* in FECD patients resulted in mutant proteins that accumulated in the ER [47]. Moreover, the LOXHD1 proteins aggregate in corneal cells carrying *LOXHD1* mutations in FECD [13]. Analysis of the corneal endothelium from the FECD patients showed enlargement of rough ER and upregulated UPR markers, including the α subunit of the eukaryotic initiation factor 2 (eIF2α), a glucose-regulated protein and a C/EBP homologous protein (CHOP) [55]. TGF-β signaling in the CECs of FECD contributes to the abnormal accumulation of ECM protein in the ER, eventually leading to the formation of unfolded protein and resulting in apoptosis via UPR [23]. One study demonstrated that the deposition of unfolded protein continuously stimulates ER stress, which in turn activates UPR. When the UPR cannot balance with the unfolded protein, it triggers the apoptosis mechanism via three signal transducers: activating transcription factor 6 (ATF6), pancreatic endoplasmic reticulum kinase (PKR)-like endoplasmic reticulum kinase (PERK) and inositol-requiring enzyme 1 (IRE1) (Fig. 1a) [56]. ATF6 is transferred to the Golgi body, where it is hydrolyzed by the Golgi site-1 and site-2 proteases, which subsequently activates the ATF6. Activated ATF6 leads to an increased activity of the CHOP protein (Fig. 1a) [57], which is also activated by activated PERK via phosphorylation of eIF2α (Fig. 1a) [58]. The activation of CHOP can lead to apoptosis [59] and down-regulates B-cell lymphoma-2 (Bcl-2) known as anti-apoptotic protein (Fig. 1a) [60]. Phosphorylation of the c-Jun N-terminal kinase induced by IRE1, subsequently inhibits antiapoptotic genes such as Bcl-2, and thus inhibits apoptosis (Fig. 1a) [61]. Meanwhile, ER stress stimulates the release of Ca^2+^, which comes into the mitochondria, contributing to mitochondria producing more ATP. At the same time, more reactive oxygen species (ROS) are produced, when the increase of ROS level exceeds a certain threshold, mitochondria will release the mitochondrial cytochrome c, which can activate the apoptosis of CECs by caspase-9 and caspase-3 (Fig. 1a) [62].

#### Oxidative stress

The corneal endothelium is particularly vulnerable to oxidative stress because it is exposed to light and vigorous metabolic activity caused by a high oxygen demand. Increased nitrotyrosine, a by-product of ROS, suggests that oxidative damage indeed occurs in FECD [63]. Because CECs are rich in mitochondria when they are stopped after mitosis, they are highly susceptible to oxidative damage due to their metabolically active pump and barrier functions. Peroxiredoxin are antioxidants that are anti-apoptotic, and Prx-2, -3, -5, and -6 are expressed in different parts of human corneal endothelial cells. A proteomic study confirmed the downregulation of peroxiredoxin (Prx-2, -3 and -5) in corneal endothelia, hinting at an incremental susceptibility to oxidant-induced damage [64]. NADPH quinone oxidoreductase 1 (NQO1) is a highly inducible and cell-protective flavoprotein that restrains the occurrence of ROS and free radicals in a cellular condition [65]. The expression of NQO1 is up-regulated by the nuclear factor erythroid 2-related factor 2 (Nrf2) transcription factor via binding to the sequence of antioxidant reaction elements in the upstream promoter region of NQO1 [66–68]. The antioxidant response related to protective Nrf2 is significantly decreased, resulting in the oxidant-antioxidant imbalance that characterizes FECD [66]. A recent report detected that the NQO1 protein levels in FECD specimens down-regulated quite dramatically, a reduction that was further confirmed in FECD patient-derived immortalized cell lines that presented with oxidative DNA damage [69]. The overexpression of NQO1 significantly decreases ROS levels and DNA damage caused by menadione (MN) and catechol estrogen stressors [69]. Moreover, Liu et al. [70] established a nongenetic FECD animal model by exposure to ultraviolet A, which caused greater mitochondrial DNA (mtDNA) and nuclear DNA damage in female mice. The sex-dependent effect of UVA was driven by the activation of estrogen-metabolizing enzyme CYP1B1 and formation of reactive estrogen metabolites and estrogen-DNA adducts in female but not male mice, causing CYP1B1-mediated estrogen genotoxicity. Together, these data confirm that an oxidant-antioxidant imbalance in FECD can induce oxidative DNA damage and apoptosis.

#### Mitochondrial dysregulation pathway

Mitochondria, found in eukaryotic cells, are organelles covered by two membranes. They are the main site of ATP production within the electron transport chain. In addition to energy supply, mitochondria participate in other cell processes such as regulating calcium levels and apoptosis. The cornea is highly exposed to external elements; it receives a large amount of atmospheric oxygen and sunlight, including ultraviolet light, which prompts ROS generation in the cornea, mitochondrial dysfunction and oxidative damage to the cells. Furthermore, due to its post-mitotic nature, the corneal endothelium tends to accumulate mtDNA damage. Corneal tissue from FECD patients revealed an increase in ROS and 8-hydroxy-2′-deoxyguanosine (both of which are signs of oxidative damage) mainly in the mtDNA of CECs gathered around the rosettes [66]. In one study, FECD patients presented with significantly more mtDNA damage and lower DNA repair efficacy compared to normal controls [71]. Compared with normal corneal specimens, FECD human corneal endothelial cell lines and FECD CECs showed extensive mtDNA and nuclear DNA damage, as measured via quantitative polymerase chain reactions [72]. Subsequent studies increased endogenous cellular oxidative stress with MN, a quinone metabolized by a 1-electron reducing enzyme to produce intracellular superoxide and an unstable semiquinone radical that increases intracellular ROS, to model the pathognomonic rosette formation-a characteristic morphological change of FECD. MN induced rosette formation and damaged the mtDNA and nuclear DNA, which were rescued with N-acetyl-cysteine pre-treatment [72]. One study featuring the FECD explants reported that mtDNA levels increased and telomeres shortened [73]. FECD does not evenly affect the integrity of CECs. Some of the surviving FECD CECs were in a compensatory state of function. The surviving CECs may compensatively increase the mitochondrial content and produce ATP required by the Na/K-ATPase ion pump to maintain the relative dehydrating state of the corneal stroma. Therefore, the level of mtDNA is increased in the surviving FECD CECs. Interestingly, cell culture can rehabilitate mtDNA levels, telomere length, oxidant-antioxidant gene expression balance, and sensitivity to oxidative stress-induced cell death. Therefore, choosing the more functional FECD CECs for cell culture may provide a basis for future treatments [73]. Méthot et al. [74] studied a series of events leading to mitochondrial exhaustion as follows: FECD CECs initially helped to generate more ATP by adding mitochondrial calcium to compensate for endothelial cell loss, leading to increased oxidation. The CECs then increased mitochondrial mass, mitochondrial calcium and mitochondrial membrane potential in response to the continuous demand for more ATP. In this phase, the CECs peaked at their maximum capacity and began to respond to irreversible oxidative damage leading to mitochondrial burnout, causing cell death via apoptosis. Meanwhile, the mitochondrial membrane potential decreased, and calcium was released from the mitochondria.

#### Apoptosis pathway

Apoptosis, the spontaneous and orderly death of genetically controlled cells, is characterized by cell shrinkage, membrane blebbing, chromatin condensation and DNA fragmentation [75]. Apoptosis is considered to be an important mechanism of FECD. Analysis of endothelium flatmounts showed apoptotic CECs in the FECD group, and the control group showed no apoptotic cells. Further, the observed percentage of apoptotic endothelial cells was much higher in the FECD group compared to the controls. These are the first findings that demonstrate that apoptosis plays a significant role in endothelial cell death in FECD [76]. Subsequently, intense Fas, FasL and Bax staining were found in FECD patients, and faint staining of Bcl-2 was observed occasionally in FECD patients, all of which points to a disturbance in apoptotic regulatory molecules [77]. Eleven of the 14 corneas with FECD showed positive TUNEL labelling, indicating the activation of apoptosis [78]. A recent study detected mitochondrial dysfunction, which can lead to cytochrome c release and subsequent caspase breakage, promoting cell death by apoptosis [72]. Hence, these studies prove that apoptosis is closely related to FECD pathogenesis. Previous studies suggest that CECs’ death may also be caused by other mechanisms. For example, apoptotic CECs in FECD show an increase in mitochondrial Ca^2+^ [74]. A massive and/or a prolonged accumulation of Ca^2+^ in the mitochondria can lead to the release of cytochrome c, which drives the activation of caspase-3 [79]. Therefore, an increase in mitochondrial Ca^2+^ of FECD CECs probably promotes apoptosis (Fig. 1b). Moreover, SLC4A11, localized at the basolateral surface of CECs, contributes to osmotically-driven water flux from the stroma to aqueous humor to maintain the relative dehydration of the cornea [80], and four mutations associated with corneal endothelial dystrophy can impair solute transport (water flux) function [80, 81]. Therefore, *SLC4A11* gene mutations potentially result in FECD CECs edema and rupture (Fig. 1b).

#### Mitophagy

Autophagy is a cellular process featuring ER stress and oxidative stress. Forming autophagosomes and combining them with lysosomes, autophagosomes phagocytose the resulting substance and the organelles in the cell. Autophagy plays an important protective role in cells. However, autophagy defects or overactivation can lead to cell death [82]. In animal models of FECD, DNA-damage regulated autophagy marker 1 was up-regulated [54]. Lithium therapy increased autophagy in mice subjects with FECD, possibly contributing to enhanced endothelial cell survival [83]. Mitochondrial health is determined by mitochondrial quality control namely, fission, fusion and mitophagy [84]. When mitochondrial fission and fusion occurs in repetitive cycles but cannot reduce mitochondrial damage, mitophagy is activated to clear the mitochondria [84]. Increased numbers of autophagic vacuoles were found in FECD tissues containing degraded and swollen mitochondria with cristolysis [85]. In the same study, the elevated autophagosome components (microtubule-associated protein 1 light chain 3-II and lysosomal-associated membrane protein 1) and the downregulation of mitochondrial fusion protein mitofusin 2 in mitochondrial fractions suggested a loss of mitochondrial fusion ability; here, fragmented mitochondria entered the pre-autophagic pool and activated autophagy [85]. Furthermore, in FECD, intracellular oxidative stress induces Parkin-mediated mitochondrial fragmentation whereby endogenous Dynamin-related protein 1 and PTEN-induced putative kinase 1 are segregated for degradation via mitophagy in the process of degenerative cell loss after mitosis of ocular tissue [86].

#### Epithelial-mesenchymal transition pathway

EMT is not only associated with embryonic development, but it is also related to wound healing, organ fibrosis and tumor occurrence and development [87]. The up-regulation of EMT-related genes *ZEB1* and *SNAI1* by TGF-β in CECs in FECD patients was associated with the deposition of ECM proteins [88]. Furthermore, the TGF-βR1 inhibitor (SB431542) suppressed the expression of *ZEB1* and *SNAI1*, leading to a decrease in ECM. This suggests that blocking the TGF-β signaling pathway is helpful for FECD treatment [88]. A study using an *in vitro* model of FECD provided the first evidence that oxidative stress induced by MN resulted in EMT, leading to increased expression of *SNAI1*, *ZEB1*, fibronectin and N-cadherin in CECs [89]. The overexpression of both isoforms of *E2–2* factors (E2–2A and E2–2B) in MDCK cells can induce EMT [90]. *Snail1* up-regulates *E2–2* expression, which up-regulates *ZEB1* expression [24], suggesting that mutations in both *TCF4* and *TCF8* may share a common pathologic pathway. Moreover, decreased expression of miR29 family members in CECs from FECD may increase the deposition of ECM, including collagen I, collagen IV and laminin [91, 92].

#### RNA toxicity and repeat-associated non-ATG translation

Wieben et al. [93] were the first to report that the corneal endothelium from FECD patients harbored a unique signature of mis-splicing events caused by CTG TNR expansion in the *TCF4* gene. They demonstrated that TNR expansions in the *TCF4* gene lead to FECD through a mechanism associated with sequestration of muscleblind-like protein 1 in the RNA foci. The length of the CTG triplet repeat allele seems to be associated with disease severity [35]. Furthermore, TGC repeat lengths > 50 was found in up to 79% individuals with FECD; they were found in only 3% of unrelated individuals, suggesting that trinucleotide amplification may predict disease risk [31]. Interestingly, Foja and colleagues [94] reported the CTG repeat expansion may reduce gene expression of *TCF4*. By contrast, Okumura and colleagues [95] reported that *TCF4* mRNA is upregulated in FECD CECs, regardless of the presence or absence of TNR expansion, but the length of the TNR in cases with expansion tended to be positively correlated with *TCF4* expression level. According to a recent study, the levels of *TCF4* transcripts change bidirectionally in response to an expanded CTG TNR; a decrease of *TCF4* expression of proximal downstream promoters linked to these 5′exons while an increase in the levels of *TCF4* transcripts encoded by downstream alternative 5′exons distal to the CTG TNR, possibly indicating a compensatory mechanism, explaining why previous studies on the level of *TCF4* transcripts in FECD showed different results [96]. Almost all of the TNR expansion diseases thus far have been directly related to rare neurologic or neuromuscular diseases. Among eye diseases, FECD was first found to be related to TNR expansion. The expanded CTG·CAG repeat initiates transcription and translation through non-ATG in the third intron of *TCF4*, which provides a basis for studying repeat-related non-ATG translation in the CECs of FECD patients [97]. RNA focal points co-localized with the splicing factor muscleblind-like protein 1 in CECs from FECD patients; mRNA splicing changes also occurred. Combined, these represent the first evidence of RNA toxicity and mismatch in common non-neuro/neuromuscular diseases associated with repetitive expansion [98].

#### Other pathogenesis

A recent study found that SLC4A11 is a cell adhesion molecule, mediating CECs adhesion to DM. Four FECD-causing mutations in SLC4A11 extracellular loop 3 (Y526C, T561M, S565L and V575M) lead to the destruction of the adhesion of CECs to DM, which may explain the loss of CECs in FECD patients [99]. Moreover, a study demonstrated that DNA methylation alterations are crucial to the pathogenesis of FECD [100]. This study showed that promoter DNA hypermethylation of *SLC4A11*, which is critical to water transport in FECD CECs. Promoters of genes involved in cytoskeletal organization, which plays an important role in the barrier integrity of the corneal endothelium and restrict fluid leakage into the corneal stroma, tend to be hypomethylated in FECD. Promoter DNA hypermethylation of genes involved in cellular metabolism plays an important energy metabolism role in FECD CECs. All of the DNA methylation changes in genes, associated with cytoskeletal organization, cellular metabolism, and ion transport occurred in FECD CECs, may contribute to the loss of corneal transparency in FECD through changing corneal endothelia biological processes [100]. Therefore, drugs targeting DNA methylation can be developed and used for FECD treatment.

### Current therapeutic modalities

In the past few decades, penetrating keratoplasty has been an effective treatment option for FECD. However, with the innovation of surgical techniques, lamellar keratoplasty effectively utilizes limited corneal specimens. Endothelial keratoplasty has offered some distinguishing benefits such as better vision recovery, less damage to the corneal structure and reduced incidence of bleeding, infection and endothelial rejection. Endothelial keratoplasty includes Descemet-stripping endothelial keratoplasty [101], Descemet membrane endothelial keratoplasty [102] and Descemetorhexis without endothelial keratoplasty [103].

### Future therapies

#### Cell-based therapy

Corneal cell therapy is a new treatment strategy for FECD; it refers to the *in vitro* culture and expansion of primary human corneal cells before transplantation. Compared with existing healthy cells, this cell culture can restore the molecular phenotype associated with oxidative stress by selecting the more functional FECD cells [73]. In this treatment, the operator injects human CECs that were cultured *in vitro* into the anterior chamber. The patient then performs different maneuvers, such as assuming the prone position, to promote adhesion of the CECs to the DM [104]. A recent significant study confirmed the therapeutic effect of corneal cell therapy in patients with bullous keratopathy that was mainly caused by FECD [105]. The study reported that injecting cultured human CECs into the anterior chamber successfully reversed corneal edema; the clinical results were stable in the 2 years following the operation.

#### Gene therapy

FECD is typically a sporadic disease, but it can also take the form of autosomal dominant inheritance [2, 3]. Despite FECD being a genetically heterogenous disease, most humans with FECD, at least among Caucasian patients, have a CTG TNR expansion sequence in chromosome 18q21 of *TCF4* [106]. The mutant *TCF4* transcript accumulates with the repeated amplification of CTG, and its pathological effects reflect the cumulation of RNA lesions and the isolation of RNA splicing factors in the nucleus. This is similar to myotonic dystrophy-1, which is a disease associated with TNR. Researchers must keep exploring effective treatments for these diseases. For example, in muscular dystrophy-1 cell lines, inactivated Cas9 can prevent the transcription from TNR amplification. Deactivated Cas9 enzyme (dCas9) could be designed to efficaciously connect with the trinucleotide DNA repeat sequence of myotonic dystrophy type 1 cells, and thus restrain the transcription of amplified mRNA molecules [107]. Further evidence suggest that these dCas9 molecules are able to complex with pathological elongated mRNA molecules and ameliorate deleterious effects, especially in short and intermediate repeat lengths [107]. All of the studies indicated that similar dCas9 strategies, especially targeting CECs through intracameral delivery, may be able to effectively tackle the genetic variation of FECD and restore its normal phenotype.

#### Other therapies

In FECD, oxidative stress causes excessive endothelial cell apoptosis [66]. Consequently, a potential therapy for FECD is to target this pathway. N-acetyl-cysteine, an antioxidant and free radical scavenger, has been shown to rescue CECs exposed to oxidative stress and ER stress not only *in vitro* but also among *in vivo* animal models with FECD [72, 89, 108]. Research on Nrf2-related antioxidant defense deficiency provides a basis for investigating whether Nrf2 stimulator play a cellular protective role in FECD [66]. Sulforaphane, a natural glucosinolate, was found in green cruciferous vegetables [109]; it has a cytoprotective function, can increase the nuclear translocation of Nrf2, reduce the production of ROS and up-regulate several antioxidants, and thereby reduce CECs apoptosis [110]. Furthermore, Nrf2 levels can be reinforced by many other compounds (e.g., 3H-1,2-dithiole-3-thione), which intervenes in Nrf2 degradation [111, 112]. Meanwhile, TGF-β, an important regulator of EMT, is up-regulated in FECD, inducing the deposition of ECM proteins and resulting in apoptosis via the UPR in FECD [23]. Inhibition of TGF-β can suppress aggregation accumulation and the UPR as well as the activation of apoptosis [23]. Moreover, a recent study identified a non-steroidal anti-inflammatory drug, glafenine, which can correct cell surface trafficking defects in some *SLC4A11* mutants, leading to increased SLC4A11-mediated water flux in cells expressing the treated mutants, providing a framework for future personalized medicine approaches to correct SLC4A11 misfolding mutants present in FECD CECs [113]. Further studies are needed to demonstrate whether these approaches are feasible therapy options for FECD patients.

## Conclusions

This review reveals that our understanding of the pathogenesis of FECD and the development of molecular genetics is becoming more profound. The gene mutation site has been established and the molecular mechanisms are becoming clearer. However, many questions regarding the pathogenesis remain elusive. Overall, the data from the studies included here illuminate the molecular mechanisms associated with FECD and may help to optimize various therapeutic approaches.

## Data Availability

Not applicable.

## Abbreviations

FECD: Fuchs endothelial corneal dystrophy
*COL8A2*: Collagen Type VIII Alpha 2 Chain
*TCF4*: Transcription factor 4 gene
*TCF8*: Transcription factor 8 gene
*ZEB1*: Zinc finger E-box binding homeobox 1
*LOXHD1*: Lipoxygenase homology domains 1
*SLC4A11*: Solute carrier family 4 sodium borate transporter member 11
*AGBL1*: ATP/GTP binding protein like 1
ER: Endoplasmic reticulum
CECs: Corneal endothelial cells
ECM: Extracellular matrix
TGF-β: Transforming growth factor-β
SNPs: Single-nucleotide polymorphisms
UPR: Unfolded protein response
PERK: Pancreatic endoplasmic reticulum kinase (PKR)-like endoplasmic reticulum kinase
ATF6: Activating transcription factor 6
IRE1: Inositol requiring enzyme 1
eIF2α: α subunit of eukaryotic initiation factor 2
CHOP: C/EBP homologous protein
S1P: Site-1 protease
S2P: Site-2 protease
Bcl-2: B-cell lymphoma-2
JNK: c-Jun N-terminal kinase
ROS: Reactive oxygen species
NQO1: NADPH quinone oxidoreductase 1
Nrf2: Nuclear factor erythroid 2-related factor 2
mtDNA: Mitochondrial DNA
EMT: Epithelial-mesenchymal transition
TNR: Trinucleotide repeat
DM: Descemet’s membrane
dCas9: Deactivated Cas9 enzyme
